# Health-care encounters create both discontinuity and continuity in daily life when living with chronic heart failure—A grounded theory study

**DOI:** 10.3402/qhw.v10.27775

**Published:** 2015-07-01

**Authors:** Malin Östman, Eva Jakobsson Ung, Kristin Falk

**Affiliations:** 1 Institute of Health and Care Sciences, Sahlgrenska Academy, University of Gothenburg, Gothenburg, Sweden; 2 Närhälsan Källstorp Health Centre, Trollhättan, Sweden; 3 University of Gothenburg Centre for Person-Centred Care (GPCC), Sahlgrenska Academy, University of Gothenburg, Gothenburg, Sweden

**Keywords:** Chronic disease, heart failure, illness, patient perspective, continuity, person-centred care, grounded theory method

## Abstract

Living with chronic heart failure (CHF) often involves lifelong contact with health care, more or less frequently, depending on fluctuating health-generating disruptions in everyday life. To reduce the influence on continuity in life, health-care professionals should preferably focus on supporting patients in managing their daily lives, based on their perspective. The aim of this study was to describe how the interaction in health-care encounters contributes to either continuity or discontinuity in the daily life for persons with CHF. Interviews with 18 participants were carried out, using the grounded theory method, through data collection and analysis. Two core concepts were constructed from data which reveal a model that illuminates the characteristics of the encounters, the actions of health-care professionals and the normative discourse. *Patient-centred agenda* consists of the categories: *“Experiencing a subordinate approach,” “Objectifying during the encounter”* and *“Expected to be compliant.”* This describes how health-care professionals enhance discontinuity in daily life by using a paternalistic approach in the encounter. *Person-centred agenda* consists of the categories: *“Experiencing an empowering approach,” “Person-centredness during the encounter”* and *“Expected to be capable*.” It describes how participants perceive that health-care professionals enable them to deal with everyday life which enhances continuity. The findings highlight the importance of health-care professionals’ attitudes and communication in encounters with patients. Health care must be designed to support and promote patients’ own strategic thinking by strengthening their self-image to enhance continuity in everyday life. The experience of discontinuity is based on the prevailing health-care culture which focuses on disease and medical treatment and regards it as superior to the illness experience in an everyday life context. We therefore strongly suggest a paradigm shift in the health-care organisation and culture in order to support the patients in their efforts to live a meaningful, rich life, in spite of the chronic illness CHF.

Living with chronic heart failure (CHF) affects health-related quality of life (Ekman, Fagerberg, & Lundman, 2002) due to the associated consequences of fluid retention, breathlessness, fatigue, limited exercise capacity and poor survival in a 5-year perspective (McMurray et al., 2012). Studies show that having CHF also entails living with an unreliable, inadequate body with troublesome symptoms that affect everyday life, health and well-being (Jaarsma et al., 2014; McMurray et al., 2012) and, as CHF is a progressive syndrome with periods of deterioration, it often results in frequent, long-lasting health-care contact and repeated acute hospitalisation (Liao et al., 2007; Mejhert et al., 2013). These health-care contacts are sometimes seen as fragmented, due to a lack of co-ordination and poor communication, which contributes to experiences of discontinuity in care, according to a study by Browne, Macdonald, May, Macleod, and Mair (2014). At the same time, however, continuity of care in the sense of co-ordination has been shown to reduce in-patient hospitalisation, emergency department visits and the number of complications (Hussey et al., 2014).

Patients appear to experience continuity of care when they are given information, feel confident and secure about the care pathway and have a relationship with a trusted clinician (Haggerty, Roberge, Freeman, & Beaulieu, 2013). Continuity of care can be viewed from either a person perspective or a disease-focused perspective, according to Haggerty et al. (2003). The term “continuity of care” has been defined in numerous ways over the decades and mostly from the patient perspective, according to a review article by Uijen, Schers, Schellevis, and van den Bosch (2012). As a result, the most common way to study continuity of care is to measure the number of health-care contacts in relation to the physician–patient relationship over time (Jee & Cabana, 2006; Reid, Haggerty, & McKendry, 2002; Saultz, 2003).

Several studies show that the importance of continuity of care is valued more by patients suffering from chronic diseases than by patients who are relatively healthy and rarely need to seek care (McCormack, Mitchell, Cook, Reed, & Childs, 2008; Nutting, Goodwin, Flocke, Zyzanski, & Stange, 2003; Pandhi & Saultz, 2006). This is not surprising considering how patients with CHF feel that the lack of help and support from health care results in disruptions to their lives and subsequently to their being unable to live their lives to the full (Davidson, Dracup, Phillips, Padilla, & Daly, 2007; Nordgren, Asp, & Fagerberg, 2007; Yu, Lee, Kwong, Thompson, & Woo, 2008). This point of view can be linked to studies that describe how people with chronic illnesses, such as chronic kidney disease, lung cancer and stroke, experience continuity despite life-course disruptions (Becker, 1993; Leveälahti, Tishelman, & Öhlén, 2007; Llewellyn et al., 2014). They do this by using various actions, such as creating explanations, lowering their expectations and identifying markers of continuity to maintain continuity in life.

Health care in western countries generally focuses on the patient's physical problems (Naylor, 2012) and the care of patients with CHF is no exception. This biomedical dominance overlooks the integration of the patient's perspective, which impairs the health of chronically ill patients (Lindsey, 1997). When health-care professionals instruct patients in how to take care of themselves and act in relation to the disease by mainly focusing on their own assessment, according to Rabelo, Aliti, Domingues, Ruschel, and Brun (2007), this affects the patient's understanding, thereby reducing his or her ability to participate and make decisions about his or her own health (Strömberg, 2005).

The aim of this study was to describe how the interaction in health-care encounters contributes to either continuity or discontinuity in the daily life for persons with CHF. The theoretical perspective of symbolic interactionism, assumes that a person construct selves, society and reality in interaction with other persons (Blumer, 1969; Mead, 1934) due to the fact that persons are active, creative and reflective (Charmaz, 2006), underpins this study and is used as a sensitising theoretical framework for data collection and analysis.

## Method

In this qualitative study, the grounded theory method (GTM) was used to identify processes and actions inductively (Charmaz, 2009) in order to deepen our understanding of what takes place in the specific area of health-care encounters, using a systematic approach to collecting and analysing data and, furthermore, constructing concepts and a formative theory grounded on the interviewed persons’ narratives (Bryant & Charmaz, 2007; Charmaz, 2006; Glaser & Strauss, 1967).

### Participants and settings

The participants were purposively selected from three different settings: primary health-care settings, a specialist clinic at a county hospital and a local heart and lung association (HLA). The selection of settings was made to capture different phases of the disease, treatment and a variety of patient experiences of living with CHF to obtain the broadest possible picture. The severity of CHF varied from those who lived almost symptom free to those who suffered from severe physical limitations with shortness of breath and fatigue. The sample consisted of eight men and five women recruited from primary health care and a specialist clinic, added five women from the HLA. The participants had lived with CHF from 6 months to more than 5 years and their mean age was 76 years (range from 62 to 88 years). All of them had retired and 13 of them were cohabiting, while five lived alone. We included persons diagnosed with CHF, aged ≥20 years, who understood and spoke Swedish and lived in standard housing. The diagnosis of CHF was verified from the participants’ medical records, in accordance with the criteria from ESC guidelines (McMurray et al., 2012).

### Data collection

Data were collected between February 2011 and September 2012 and were extended to include four individual follow-up interviews in June 2014 to clarify and extend the previous information. The participants were sent an information letter and, after 1 week, they were contacted by telephone regarding a request for participation, the aim of the study and an appointment for an interview. All the interviews were audiotaped and, at the end of each interview, the participants were asked to give their permission to be contacted again if something needed to be clarified. All the participants gave their informed consent. After each interview, the data were transcribed verbatim and the analysis began. Individual interviews were used to allow participants to verbalise their experiences in a private interview situation. Telling their stories helped them to be aware of their own thoughts, feelings and actions. The individual interviews were conducted in the participants’ homes or in a primary care setting, according to the participants’ wishes, and lasted for 25–75 minutes (40 minutes on average). The individual interviews were the main source of data collection, but a group interview with five women was used as supplementary data. This conversation contributed to a fuller narrative, which enriched our understanding in that the participants collectively interpreted their experiences. The group interview lasted for 1 hour and 40 minutes and took place in the HLA meeting room.

An interview guide was used to support the data collection and structure the interviews on continuity and discontinuity in life. The interviews began with broad-based, open questions covering the main areas of interest. Please tell me what it is like to live with CHF. Please tell me how you make your life cohesive, despite illness. Please tell me how health-care encounters could help you to make your life more cohesive. The participants were encouraged to talk freely and there was a flexible use of the interview guide due to the participants’ narratives, because some spoke freely and some were more reticent. The participants’ stories about living with CHF and, together with the interviewer's experiences as a nurse in primary health care, guaranteed substantial, relevant data. This publication is part of a study of continuity in life, where the set of collected data contributed to two manuscripts with different aims.

### Data analysis

Data collection and coding were carried out simultaneously throughout the entire process to explore, deepen and refine questions which prompted new questions. Using a constant comparative method to systematise and analyse data, we compared meanings and identified similarities and differences (Charmaz, 2006, 2009; Glaser & Strauss, 1967). The constructions of codes, categories and concepts were carried out continuously to be more specific until no further insight was obtained (Charmaz, 2006; Morse, 2000). The texts were read thoroughly to acquire a comprehensive understanding of the whole. Open coding then began by reading the texts line by line, as well as labelling codes relating to psychosocial processes and actions that facilitated and restricted continuity with phrases or names as close to the data as possible. The codes were compared with one another, based on similarities and differences. When all the codes had been identified and began to stand out, the next step, focused coding, took place and data were organised, grouped temporarily as categories. During the entire process, codes and categories were compared and discussed by the authors and, step-by-step, in line with the constant comparative method, the analyses were brought to a more abstract level that generated concepts, confirmed by their properties. Memos based on reflections, theories and ideas were written down during the whole process. The memos helped us to construct new ideas and questions which helped us to identify gaps in data collection but also to clarify relationships between codes and categories (Charmaz, 2006).

### Ethical considerations

The study was approved by the regional Ethical Review Board in Gothenburg (Dnr.543-10) and we followed the principles outlined in the Declaration of Helsinki (WMA, 2013). Both verbal and written information regarding the aim, procedures and contact details was given to the participants. For those who wished to participate in the study, written informed consent was obtained before the interview began. The participants received information about their right to withdraw at any time, that the interviews would be recorded and transcribed and that identifying details would be removed to ensure confidentiality. Respect was paid during the interviews to the participants’ condition and, if unpleasant experiences occurred at, or after the interview, they were offered telephone contact with a counsellor for further follow-up.

## Results

**Figure 1:**
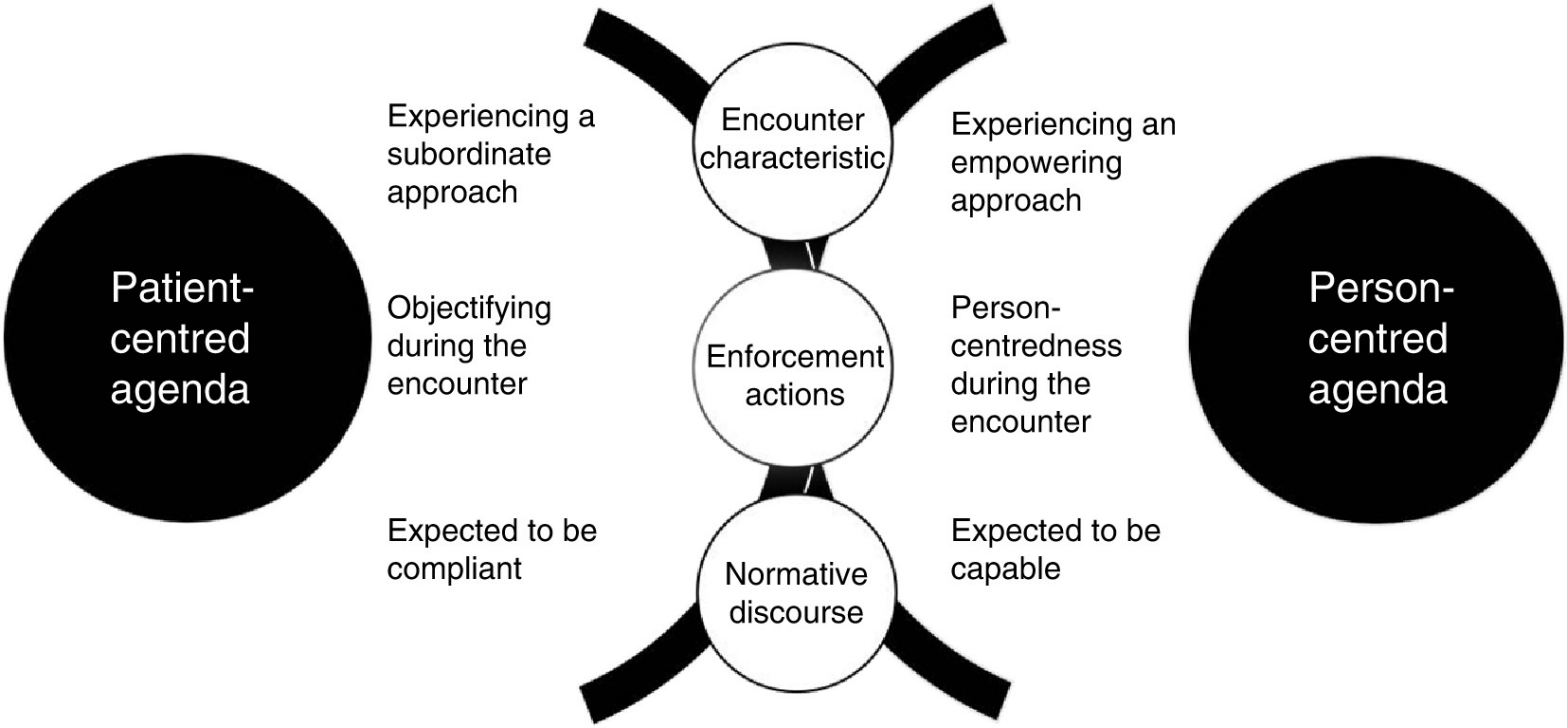
The connection between the health-care encounters experienced as enhancing discontinuity or continuity in daily life.

The findings are based on the participants’ experiences of the care encounter with health-care professionals and the descriptions include meetings with various professional representatives and various forms of health care. The health-care encounters are described by the participants as good or bad in relation to their extended experiences of continuity and discontinuity in life. These experiences are not solely based on emotions. They also illustrate rational actions and health-care environments, which are characterised by unspoken norms and procedures in the relationship between the patient and health-care professionals. The results have been summarised in two core concepts, *patient-centred agenda* and *person-centred agenda*. A tentative model illuminates the characteristics of the care encounters, the actions of health-care professionals and the normative discourse, see Figure 1. The results are presented below, with depersonalised quotes from the interviews.

### Patient-centred agenda

*Patient-centred agenda* summarises the categories “*Experiencing a subordinate approach,” “Objectifying during the encounter”* and *“Expected to be compliant,”* all of which contribute to the feeling of discontinuity, transferred from the health-care situation to the participants’ everyday life. The focus of the professionals when it comes to both expert knowledge and a preferential right to interpretation is a body with a diagnosed disease. Disease-related explanations are perceived as being superior to illness experience in the health-care situation, even if the participants’ problems and questions are based on their everyday lives. When the health-care professional mainly focuses on procedures and standards in the encounter, without taking the patient's wishes and needs into account, it becomes more difficult to continue living as before. Contacts with health care represent a break in the participants’ lives and, when the participants perceive that their everyday lives are of no interest to the health-care professionals, it generates frustration and powerlessness. Being treated as inferior and being limited against one's will are perceived as insulting and degrading. The consequence of these interruptions is a reduction in the patients’ ability to manage their everyday lives.

### Experiencing a subordinate approach

The subordinate approach describes the characteristics of the encounter with the health-care professional's attitudes, including expert role and precedence over preferential rights to interpretation. In these situations, the health-care professionals act as experts and know best, their knowledge is the prevailing factor and the participants’ perceptions and experiences are of subordinate value when it comes to care and treatment.

The health-care professionals’ attitude sometimes alienated and prevented the patients from being part of their own care. This in turn created a large number of unanswered questions about how to deal with symptoms, functional impairments and medication in their daily lives. One of the women described the experience thus:

Something that I personally missed was when the health-care professionals did not tell me about more than the symptoms or side-effects I had right then. But I want to know everything related to this heart failure and then I can weed out the things that do not apply to me. And I understand why they do not say it, it is probably that people are worried about something they are not affected by and are waiting to develop it, but I would feel much better and have a better quality of life if I knew. (Interview 1)

The participants described ways the health-care professionals used their superior role to control the encounter. When the participants express thoughts, ideas or experiences relating to their situation, they are met by a lack of interest or are questioned. The feeling that the health-care professionals are not at all interested in attempting to understand their situation contributes to a sense of helplessness and builds dependence on health care, as one of the male participants said:

There was a doctor there, who looked at my heart and he said that it works, of course. But I told the nurse that I wanted another doctor, I wanted a second opinion. A very long time passed, until late autumn, before I was given an appointment. Then the doctor came in and asked how things were and I said I was out of breath and he listened, a little behind and slightly forward. So he removed three drugs and I had to agree. It was no good, that's what I call getting fired from this place and I told him so. Yes, they can now attend to you at the health-care centre, I thought that was shameful and dreadful, especially as I had expected to get a new appointment there. They completely ignored me and just wanted to get rid of me! (Interview 6)

### Objectifying during the encounter

The participants felt that they were not treated as valuable persons in the encounter due to the health-care professionals mainly focusing on different actions and treating them as objects, like someone in a crowd. The reception could be both respectful and correct, but the relationship was characterised by actions that led to experiences of being solely regarded as a sick body.

The participants registered the way the health-care professionals focused primarily on their diagnosis and physical problems. Problems that occurred in everyday life as a result of their illness were less interesting. Anything that cannot be explained medically or resolved with medication is met by a lack of understanding or ignorance, which was perceived as not being believed, of simulating or of exaggerating problems. This was described as increasing the distance between the participants and health-care professionals illustrated by the following quote:

I think it was in connection with the influenza vaccination, I had a terrible cold and I was completely “out of it” for three days and I had a night, which felt as if my time was near. So I was really bad and I called 112. They sent an ambulance, I had to wait a while, a couple of hours and felt how everything subsided. When I went in, the girl in the ambulance took some samples and concluded that there was nothing wrong with me. Yes, I heard when the girl in the ambulance explained that I was “faking” things. So I was lying in there on a bed and no one cared about me, they walked past out there with coffee and sandwiches in the rooms and there I lay, no one was watching me, no one. (Interview 6)

The experience of being one of many occurs when the participants are not given an opportunity to meet the same health-care professional in connection with health-care contact or that they do not know who to contact. Always having to repeat the whole of their medical history every time they meet someone new reinforces the sense that no one cares or is involved in their care and treatment. This strengthens the perception of being pushed aside and left to face your fate alone, which generates a lack of confidence in the health-care and medical service, as one woman from the group interview said.

I have my health-care centre, but what I don't have is a regular doctor, I only get to meet these young boys and girls (interns), they are new all the time. It doesn't help me! I need someone ordinary, familiar, old and experienced. Yes, anyone who knows me, that I have confidence in. (Group interview)

According to the participants, health-care professionals are only interested in following guidelines, standards and routines based on their medical condition, regardless of their needs. This contributes to the discrepancy between the patient's expectations in relation to health-care professionals’ intent regarding the encounter. When the illness progressed and it was no longer possible to improve the participants’ condition with medical treatment, responsibility for care was transferred to a lower care level. This became a reality when the health-care professionals did not take account of the participants’ wishes for continuity in health care and reinforced the experience of not receiving the necessary care and treatment. This could be expressed as follows.

It is not so easy to keep the disease away with all the medication I have today. I would like to come back for a return visit, but the doctor says my medical treatment is finished. He called me late one night and said, “I can see from your notes that you are not feeling particularly well right now. We have checked your medicines and you have been prescribed what you need.” Then he mentioned the transplantation of both lungs and heart, but told me I was too old. He didn't have to say that to me at all. (Interview 9)

### Expected to be compliant

*Expected to be compliant* is characterised by the participants’ perception that the health-care professionals have a perception in advance that they are incapable of taking care of themselves. The participants did not comply with the patient norm and they were expected to put themselves in the hands of the health-care professionals and do what they were told in order to be given adequate care and treatment.

Adapting to the norm as patient is described as an effort to fulfil the health-care professionals’ expectations of how the participants should behave and act according to the prevailing norms, standards and skills, particularly when questioning treatment or making suggestions or acting independently. As one man said:

Yes, I take between two and three Warfarin tablets a day, but I wanted to check it out, to see if it has any bearing, so I stopped taking it and that made them a little bit angry with me. (Interview 3)

As a result, the participants attempt to adapt to routines, rules and not imposing any demands in order to be accepted and rewarded with a friendly reception although it could contribute to a lack of control, insecurity and limited freedom of action on their part. A male participant described how he tried to control his weight, in the hope that he would receive his doctor's approval.

I have lost weight, 10 kg, you can see the list of weight records (shows the list of registered weights). This is before, you can see here, at this point, I weighed this many kilos and this is after hospitalisation, when I weighed this. Yes, this is what he likes, my doctor. (Interview 12)

The participants say that the health-care professionals expected compliance with care and treatment. This means that they had to follow the routines and apply a medical approach when it came to seeing and talking about their physical problems. The language that is used can be seen to be heavily characterised by the health-care professionals’ expert knowledge. The participants said that they had to learn the language of health care by giving precise descriptions of their bodily sensations and medical treatment in order to be rewarded by the interest and involvement of the health-care professionals. One of the participants described this experience in the following manner:

Well, firstly, they didn't believe me again, because women and heart attacks and the heart, they are not the same as a man, I know that! So they thought that, as a woman, I can't have it, I don't know why it has become like this. But then I really had to tell them off! I know what I have and I know that it is my heart. Then I got these examinations that proved I had angina. (Interview 2)

### Person-centred agenda

*Person-centred agenda* is summarised as “*Experiencing an empowering approach*,” “*Person-centredness during the encounter*” and “*Expected to be capable*,” which contributed to the experience of continuity that is transferred from the health-care situation to the participants’ daily lives. Their perception of continuity in the encounter with health-care professionals is largely based on being recognised as a unique person with different needs and resources. Person-centred agenda means that the health-care professionals take account of the participants’ entire situation and that experience-based knowledge and life experience are taken into consideration and used. If the health-care professionals take account of the participants’ everyday life, their opinions, resources and potential, it creates continuity, which is characterised by positive emotions and living as before. When the participants feel confident in their relationship with health-care professionals, it increases the opportunity for them to manage the consequences of heart failure in their everyday lives.

### Experiencing an empowering approach

An empowering approach encompasses the participants’ experience of being treated as an equal by the health-care professionals, where the starting point is being regarded as an expert, given help to understand and given power over the situation.

The participants explain how they understand how everything is connected and this then improves their opportunities to be prepared to take action and develop personal strategies. In this situation, the health-care professionals make an effort to make them understand, based on their own situation, needs and wishes.

This doctor was an expert at this. I could talk to her and she took the time, you know. She was very nice. She described and drew on paper and explained how things were, it was very easy-going. (Interview 10)

When the professional experts’ knowledge is included in the participants’ own understanding, it enables them to manage their everyday lives. Active participation, planning and influence over their own decisions give the participants a feeling of greater self-confidence and self-esteem. With a comprehensive understanding and knowledge, the participants were consciously and continuously able to adapt their actions on the basis of their personal perception of health and well-being in their everyday lives. For instance one woman said:

I take things very slowly in the morning, as I take nine medications at once. Then I have to run and pee very often. So I don't want to go out to do anything until noon really, if I am going to go shopping and so on. I take it easy. Yes, that's what I do, like now I am going to do some shopping afterwards and so I shall take the diuretic a little later today. (Interview 2)

### Person-centredness during the encounter

Person-centredness during the encounter comprises the health-care professionals’ interaction during the encounter, supporting the participants to help them feel recognised, acknowledged and a unique person. It contributes to experiences of being an equal, where both personal history and individual situation are taken into account.

Experiencing that the health-care professionals are receptive to their social and emotional needs, that they are genuinely interested, attentive and display the courage to become involved in their lives helps to reduce anonymity during the encounter. The sense of relief that comes from meeting the same health-care professional when visiting various health-care facilities means that their situation and medical history are already known. This generates confidence, with the starting point that they share a common past and experience, where both parties invest in the encounter. Mutual, reliable contact with the health-care professional creates a sense of security and confidence that the professional will be there when he or she is needed. As one man put it:

Twice a year, I'm down with the nurse at the health-care centre. I have called her a few times. I can just call them all at the health-care centre. I think this is really good. The nurses know exactly what my problem is and that is the feeling I have when I talk to them, they know everything about me and I do not have to repeat it all over again. (Interview 7)

### Expected to be capable

The participants actively planned actions to handle their situation and described themselves as persons who are not a burden to health care. These strategies involved applying the health-care professional's expert knowledge by measuring their weight, fluids and pulse in order to manage the drug treatment and take control of their lives. A male participant shared how he carefully monitored and recorded his weight and daily intake of fluid.

It is precisely this that I have to think a lot about what I drink. For example, I can't just go to a party, because I have to make so many adjustments. But there are no problems that can't be overcome. I still go to parties, but I make sure I only have one glass of wine. This is because of the amount of fluid, among other things (shows list of records of weight and daily intake of fluid). So, on days I am going to go to a party, I hold back on the fluid in the morning, so I can take a glass in the evening. (Interview 12)

Another strategy involves updating knowledge by searching for factual information but also by exchanging experience and ideas with relatives, friends or members of associations where the participants share a common interest. This is described as essential when it comes to feeling capable. As one participant said:

About this internet, where you can read about everything, both good and bad. I have not spent much time on the internet, but it has increased now, especially because I have been involved in these programmes and the sites on heart failure and different problems relating to it. The information there is very enlightening and I am quite interested to find out about it. (Interview 10)

Making independent decisions relating to care and treatment was another way of strengthening the participants’ picture of themselves as capable. The participants’ experience of health-care professionals having confidence in their ability to test, evaluate and reflect on their own choices and decisions helped them develop and refine action strategies. One woman put it like this.

I understand it now! Both doctors and nurses have told me that I mustn't wait at home for such a long time when I start to feel the signals, I should go immediately. Like when my appetite disappears and I feel bad and things don't work and I just want to lie down, then it is time again to go to the hospital. (Interview 2)

Another strategy involves adapting the drug treatment to symptoms and the individual situation, skipping doses, adjusting doses or stopping the treatment on their own initiative. The experience of being capable and able to make independent decisions helped the participants to remove different obstacles in their everyday lives and gave them the freedom to lead the lives they wanted. As one of the male participants commented:

After a while, I got a lot of cramping in my legs and I thought it was because of this cholesterol medication. So I simply stopped taking it, because I have never had high cholesterol and I have taken samples. I heard the nurse say that you have this medicine to paper the blood vessels, for extra protection. But I don't care! Because the cramp was so uncomfortable and it has become much, much better since I stopped taking them. (Interview 5)

## Discussion

The main finding from our results, summarised and abstracted in two core concepts, *patient-centred agenda* and *person-centred agenda*, illustrates the participants’ experience of the health-care encounter and its consequential effects on continuity in their daily life. The description of the health-care paradigm as a “patient encounter” is marked by contradictory experiences of continuity, where one side contributes to discontinuity and the other side to continuity in relation to characteristics, actions and norms. Our participants describe how the “patient encounter” leads to discontinuity to a greater extent, while the “person encounter” helps to enhance continuity in life, in that it appears to affect the persons’ balance in life, their self-image and aspirations for the future in a positive way. Although a *patient-centred agenda* can be crucial in certain emergency situations, such as brain injuries or acute cardiac events, it does not always help to enhance continuity for persons with a chronic disease such as CHF.

The core concepts of *patient-centred agenda* and *person-centred agenda* were constructed and grounded in data based on the participants’ descriptions of their experiences of continuity. The findings are presented as dichotomies that can be perceived as correct or incorrect health-care behaviour, but it was not our intention to evaluate the professionals’ actions. We have, however, chosen to describe the participants’ experiences of what contributes to continuity or discontinuity in everyday life in connection with their descriptions of what is perceived as good or bad in health-care encounters. The dichotomies help us to illustrate two sides of the same phenomenon. This is reinforced by Haggerty et al. (2013), who suggest that it may be easier to highlight what continuity means by defining what is experienced as discontinuity in the encounter with health-care professionals.

The core concept, *patient-centred agenda*, based on the categories of “*Experiencing a subordinate approach,” “Objectifying during the encounter”* and *“Expected to be compliant,”* resulted in experiences of discontinuity due to poor communication, fragmentation and competing motives in the health-care encounter. Being met by a paternalistic approach appears to reduce the participants’ ability to understand their illness and take control of their lives. This may occur because of the strong medical culture with prevailing values and beliefs which have established roles among health-care professionals and patients, where the professionals are seen as experts and decide what is best for the patients, with no regard for patient autonomy (Cribb & Entwistle, 2011; McCormack, 2003; McCormack et al., 2008). Moreover, Browne et al. (2014) found barriers to health-care quality in advanced heart failure, where both patients and professionals agreed that the current health-care environment and time constraints led to insignificant conversations. This is in line with our results, where our results also show how these health-care encounters affect the participants’ experiences of increasing discontinuity in their daily lives.

Our results relating to *patient-centred agenda* are partly consistent with the experience of uncaring encounters reported by Halldórsdóttir (1996), due to the feeling that health-care professionals are impersonal, not affected, insensitive and indifferent to the patients’ situation. This agrees with Drew and Dahlberg's (1995) study of reductionism in care when considering how patients’ experiences of illness or health are reduced to the lowest common denominator, a specific diagnosis, without respect for the patient as a whole. This kind of disheartening interaction was also found by Soelver, Rydahl-Hansen, Oestergaard, and Wagner (2014), who established that minimising conflicts was a strategy that influences the perception of continuity in basic palliative care in patients with advanced cancer. Our results, however, add another aspect that shows how the encounter with health-care professionals may be perceived as an obstacle, contributing to a problematic process of readjustment when living with a chronic illness, such as CHF. Based on these results, we can assume that health-care encounters need to be conducted differently to support patients with long-term illness compared with patients with acute illness in order to enhance continuity of life for the studied group.

The other core concept, *person-centred agenda*, helped to enhance continuity according to the categories “*Experiencing an empowering approach*,” “*Person-centredness during the encounter*” and “*Expected to be capable*.” This occurs when health-care professionals are sensitive and responsive to the participants’ experiences and regard them as a person with equally important knowledge. Supporting the sense of being an important part of one's own care and treatment gives the participant confidence in their own ability to handle future situations in life. Involvement in care is of great general importance and can be associated with the results of a meta-synthesis study of continuity of care by Waibel, Henao, Aller, Vargas, and Vázquez (2012). However, our results also show how sharing experiences with significant persons, such as familiar health-care professionals, and finding flexible strategies for dealing with various situations in daily life is of great importance when it comes to enhancing continuity. This is probably due to the importance of being in a relationship that emphasises the interpersonal process, where everything is seen as the creator and co-creator of meaning in the social world (McCormack, Karlsson, Dewing, & Lerdal, 2010; Smith, 2010).

We found that being seen as a person, with one's own features and capabilities, who develops different strategies based on an individual understanding and life horizon, contributed to confidence in being a capable person. This is in line with the capability approach reported by Sen (2005), which aims to affect quality of life by focusing on what persons can do and be. This can be coupled to a study by Nunstedt, Nilsson, Skarsater, and Kylen (2012) regarding the way people with major depression understand their illness and how they try to understand how parts and wholes relate to each other in order to manage everyday life. This confirms our results that it is not just a question of conveying knowledge within health-care encounters, as experienced as a result of the *patient-centred agenda*. Something more is needed for persons with CHF to make them regard themselves as capable of handling the illness. In this case, a *person-centred agenda* helps them to acquire an understanding and improve their ability to manage daily life and reduce unforeseen interruptions, thereby enhancing continuity in life.

The results for *person-centred agenda* have similarities to the multidimensional concepts of continuity of care according to informational and relational continuity (Haggerty et al., 2003; Waibel et al., 2012). In spite of this, surprisingly little was mentioned in the interviews about management continuity, such as transferring information or co-operation between health-care professionals. This could naturally be due to the fact that the questions during the interview failed to capture these aspects or that the patient only met a single health-care professional. However, according to Haggerty et al. (2013), the patients assume that this works and they do not reflect on the health-care structures and co-operation between health-care professionals until proven otherwise, which confirms our lack of findings of a perception of management continuity.

Our finding of being seen as a person with experiences, understanding, resources and a life story shows similarities with person-centred care, which helps patients to maintain their own identity as unique and irreplaceable through a relationship based on mutual respect (Edvardsson, Winblad, & Sandman, 2008; Ekman et al., 2011; McCormack et al., 2010). Person-centred care has also been shown to strengthen patients’ own ability to manage CHF and reduce the impact of uncertainty when patients and health-care professionals are involved together (Dudas et al., 2013). In addition, our results relating to a *person-centred agenda* demonstrate that it not only helps to reduce suffering, it also supports the process of readjustment to redefine and preserve continuity in daily life for persons living with CHF.

The word “continuity” and especially the lack of continuity is used in various studies of experiences of living with CHF, in relation to health care (Ryan & Farrelly, 2009), quality of care (Browne et al., 2014), patient satisfaction with care (Adler, Vasiliadis, & Bickell, 2010) and treatment burden, for example (Gallacher, May, Montori, & Mair, 2011). However, the concept of continuity of care has also been studied in numerous ways and has changed over time (Uijen et al., 2012), but it still appears that there is no consensus. Nevertheless, studies illuminating the continuity of care have been published frequently, but this is more a question of evaluating patients’ experiences of health-care reforms than capturing the experiences of continuity, according to Bentler, Morgan, Virnig, and Wolinsky (2014).

However, there are many different aspects of continuity and many of them can be included within the concept, but these studies have not used the concept of continuity, put into context, as we have. Our results are therefore ground-breaking in that they show the importance of being treated as a unique person with individual needs and prerequisites in the health-care encounter, even if it represents a small part of the person's ongoing life. In spite of this, these encounters affect the balance of life, the person's image of himself or herself and how to manage various situations in the encounter over time. This means that the total experience of the encounter may enhance continuity, helping to strengthen the person in daily life and give them more opportunities to live the life they desire, even if there are naturally other factors that will influence the continuity in life when living with CHF.

### Methodological considerations

According to GTM, the coding categories are created from qualitative interviews and provide a substantiation based on the participants’ experiences of the way the consequences of health-care encounters help persons to construct meaning and actions in relation to experiences of continuity when living with CHF. The research design suited our study very well and, with continuity as a sensitising concept, it was possible to answer the research question (Charmaz, 2006) about what is required in encounters with health-care professionals to experience continuity in daily life.

By describing the method and analysing in detail, according to Charmaz (2006), based on the concepts and with quotes from the interviews, we attempted to attain trustworthiness. We sought credibility by gathering rich data, making constant comparisons of codes and categories, conducting repeated critical reviews of analyses and using relevant literature, as well as the researchers’ reflexivity. The new insights into the way perceptions of health-care encounters support continuity in everyday life when living with CHF helped us to achieve originality in our study. In terms of resonance and usefulness, the findings provide new and relevant knowledge relating to the way health-care professionals’ approach can support a manageable everyday life, despite alterations when living with CHF.

One limitation of this study could be that we did not follow the participants over time, which means that we are unable to say whether there were changes in their perception of continuity across time and in relation to deterioration. Another limitation could be the high mean age (76 years) and the co-morbidity. However, CHF affects elderly persons more widely and they often live with more than one chronic disease, i.e., diabetes, vascular disease and lung disease, as can be seen in other studies (Jhund et al., 2009; McMurray et al., 2012; Yancy et al., 2013). At the same time, it may be useful to consider this group, as we know that co-morbidity and being elderly result in a desire for continuity of care according to (Bayliss, Edwards, Steiner, & Main, 2008; Nutting et al., 2003).

The choice of individual interviews with persons from different health-care contexts was made in order to obtain a broad picture of the studied area and, to complement data collection, a group interview was used with members from a local HLA. The choice of members from an association was made to gain further insights, as being in an association represents reflective persons in a modern society, who reflect on their disease and its consequences, according to Giddens (1991).

The strength of our analysis was the cross-checking between the authors. Discussions when something new or incomprehensible views occurred improved the quality of the analyses and further data collection. By working on categories and concepts grounded in the data, our findings show how health-care encounters contribute to either continuity or discontinuity in the daily lives of persons with CHF.

## Conclusion

This study highlights the participants’ experiences of health-care encounters in relation to what enhances continuity or discontinuity in everyday life when living with CHF. These results are important for both patients and health-care professionals, given that care must be designed to support patients to manage various situations related to CHF in their everyday lives. A *person-centred agenda* that involves listening to patients’ concerns, giving them support and promoting their own strategic thinking leads to the utilisation of preparedness to manage their situation in everyday life. Helping patients to attain positive strategic thinking by emphasising their functional abilities instead of focusing on the pathological course of the disease or non-working abilities is of great importance and strengthens their self-image, which in turn enhances continuity. This might be helpful in broadening the discussion and the implementation of treatment associated with CHF in order to promote health.
